# Phenotypic Features Determining Visual Acuity in Albinism and the Role of Amblyogenic Factors

**DOI:** 10.1167/iovs.65.2.14

**Published:** 2024-02-06

**Authors:** Frank A. Proudlock, Rebecca J. McLean, Viral Sheth, Sarim Ather, Irene Gottlob

**Affiliations:** 1 The University of Leicester Ulverscroft Eye Unit, Psychology and Vision Sciences, University of Leicester, Robert Kilpatrick Clinical Sciences Building, Leicester Royal Infirmary, Leicester, United Kingdom; 2 Health Sciences School, University of Sheffield, Sheffield, Yorkshire, United Kingdom; 3 Oxford University Hospitals NHS Foundation Trust, Headley Way, Headington, Oxfordshire, United Kingdom; 4 Department of Neurology, Cooper University Health Care, Cooper Medical School of Rowan University, Camden, New Jersey, United States

**Keywords:** albinism, foveal hypoplasia, visual acuity, nystagmus

## Abstract

Albinism is a spectrum disorder causing foveal hypoplasia, nystagmus, and hypopigmentation of the iris and fundus along with other visual deficits, which can all impact vision. Albinism is also associated with amblyogenic factors which could affect monocular visual acuity. The foveal appearance in albinism can range from mild foveal hypoplasia to that which is indistinguishable from the peripheral retina. The appearance can be quickly and easily graded using the Leicester Grading System in the clinic. However, interquartile ranges of 0.3 logMAR for the grades associated with albinism limit the accuracy of the grading system in predicting vision. Here, we discuss the potential role of nystagmus presenting evidence that it may not be a major source of variability in the prediction of visual acuity. We also show that interocular differences in visual acuity are low in albinism despite high levels of amblyogenic factors indicating that active suppression of vision in one eye in albinism is uncommon.

Albinism, a genetic condition characterized by reduced pigmentation in the eyes, hair, or skin, is associated with a series of anomalies along the visual pathway, including high refractive errors,^1^ iris transillumination,^2^^,^^3^ foveal hypoplasia (FH),^4^ retinal nerve fiber layer thinning,^5^ optic nerve head abnormalities,^5^ chiasmal misrouting,^6^^–^^8^ nystagmus and strabismus,^9^ and changes in connectivity in the striate and extrastriate cortices.^10^^–^^14^ All these features are likely to contribute to visual impairments, which presents a challenge in understanding the limitations on vision in people with albinism (PwA) and how they can be improved. The advent of high-resolution retinal imaging through optical coherence tomography (OCT) and adaptive optics^4^^,^^15^^–^^21^ has furthered our understanding considerably of the association between FH and best-corrected visual acuity (BCVA). However, the contribution of other visual anomalies to reduced BCVA in PwA are not well understood. In this article, in addition to FH, we explore the role of other visual phenotypical features in albinism with a view to improving the prediction of visual acuity in PwA. We focus particularly on nystagmus because this is one of the few phenotypical features in which within-subject changes can be generated by utilizing the null region characteristics of PwA.

A further aspect of visual acuity that is underexplored in PwA is monocular visual acuity. In the normal visual system, information from corresponding points in visual space for each eye are brought together in the primary visual cortex through the formation of ocular dominance columns to facilitate the detection of retinal disparities.^22^ In PwA, retinal ganglion cell axon misrouting through the optic chiasm leads to an abnormal projection of the ipsilateral visual field from the temporal retina being superimposed upon the normal representation of the contralateral visual field projecting from the nasal hemiretina, also disturbing the development of ocular dominance columns.^11^^,^^23^ In people without albinism, monocular deprivation and imbalanced refractive state (i.e. anisometropia) leads to amblyopia with selective reduction of visual acuity in one eye. These visual changes are associated with changes in the primary visual cortex connectivity with a shift in ocular dominance away from the affected eye.^24^ In contrast, recent animal studies indicate that, in the case of misaligned eyes (i.e. strabismus), suppression in an amblyopic eye is more likely to be mediated at a higher cortical level.^25^ High levels of amblyogenic factors exist in PwA but their effect on interocular differences in visual acuity has not been fully explored.^9^

All participants included in this article, whether in previously published or unpublished findings, were diagnosed with albinism using the diagnostic criteria described by Kruijt et al.^26^

## Foveal Structure and Best-Corrected Visual Acuity in PwA

**Figure 1.**
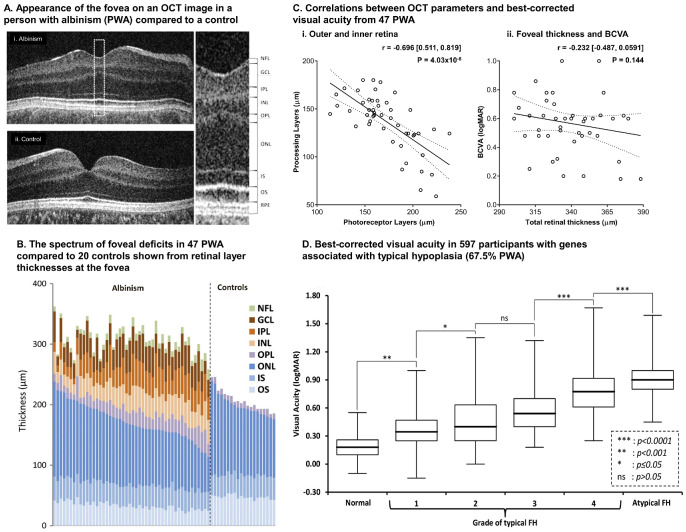
The spectrum of foveal hypoplasia (FH) in people with albinism (PwA) and the relationship between FH and best-corrected visual acuity (BCVA). Panels (**A**) to (**C**) are derived from Mohammad et al., 2011.^17^ (**A**) The appearance of the fovea in albinism from an optical coherence tomography B-scan image compared to a control; (**B**) shows the spectrum of foveal hypoplasia as indicated by the differences in retinal layer thicknesses for the inner (*orange/red colors*) and outer (*blue colors*) retina compared to controls. Continuation of all inner layers of the retina (i.e. NFL = nerve fiber; IPL = inner plexiform layer; GCL = ganglion cell layer; INL = inner nuclear layer) was common to all 47 PwA. The outer nuclear layer (ONL) and photoreceptor outer segments (OS) are the two layers of the outer retina most affected in the albinism phenotype. IS = inner segment; OPL = outer plexiform layer; RPE = retinal pigment epithelium. (**C**) Shows the relationship between: (i) inner and outer layers for PwA, (ii) the association between foveal thickness and BCVA. (**D**) From the study by Kuht et al. 2022^4^ showing box plots indicating the interquartile ranges, minima and maxima for all 4 grades of foveal hypoplasia along with no foveal hypoplasia in 597 participants with genes associated with typical hypoplasia of which 67.5% were PwA. The 95% confidence intervals for *r* values are also shown. Data from both eyes were averaged where available and BCVAs are recorded with both eyes open.

The fovea in albinism resembles that of a premature infant^27^ with a reduced or absent foveal pit, continuation of inner retinal layers across the fovea, reduced or absent specialization of the outer retina^17^ and also a reduced or absent avascular zone (Fig. 1A).^28^ PwA suffer mainly from mild to moderate visual impairment, with a wide spectrum of BCVAs ranging from around 0.2 to 1.0 logMAR.^9^ Similarly, early OCT studies demonstrated that foveal structure in PwA forms a continuum (Fig. 1B), with inner retinal layer thickening and outer retinal layers thinning being directly correlated in relation to severity of FH (Fig. 1Ci).^17^ As a result, measuring total macular thickness, which is a sum of inner and outer retinal layers and a common metric used in macular diseases, is a poor indicator of BCVA in PwA (Fig. 1Cii).

## Clinical Grading of Foveal Hypoplasia in PwA

**Figure 2.**
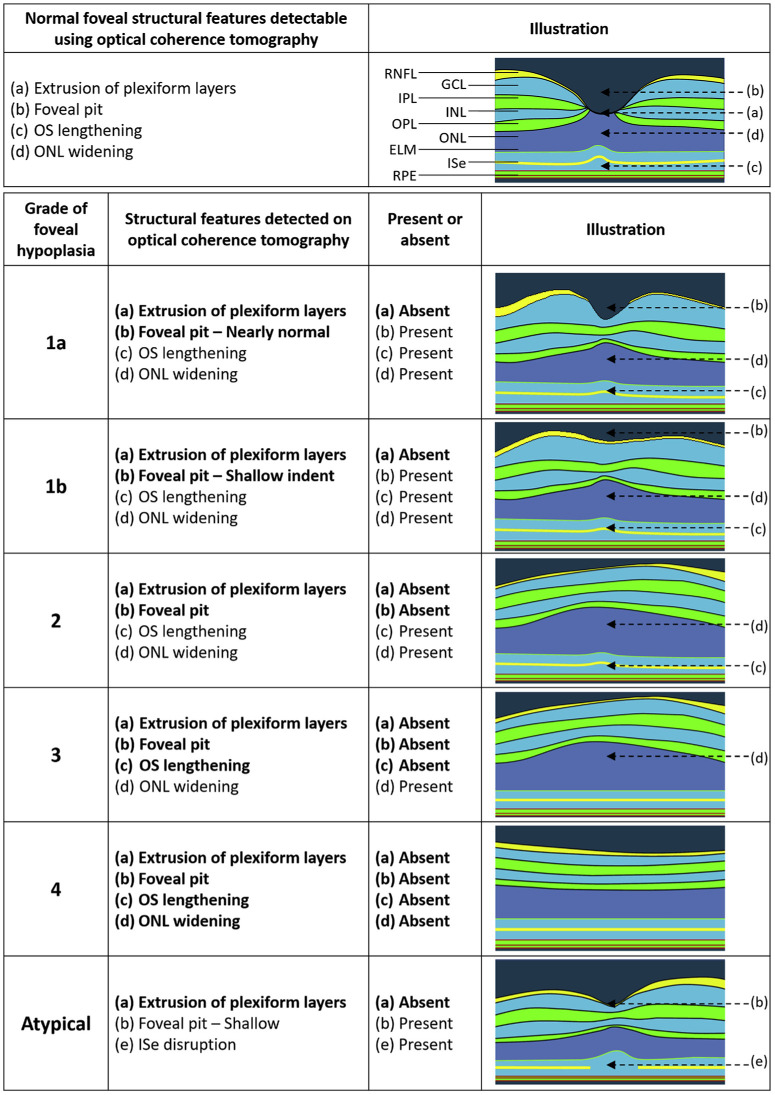
The Leicester clinical grading scheme for foveal hypoplasia (from Rufai et al., 2020).^56^ RNFL = retinal nerve fiber; GCL = ganglion cell layer; IPL = inner plexiform layer; INL = inner nuclear layer; OPL = outer plexiform layer; ONL =outer nuclear layer; ELM = external limiting membrane; ISe = inner segment of photoreceptors / ellipsoid boundary; RPE = retinal pigment epithelium.

Foveal hypoplasia (FH) can be graded clinically, without measuring individual layers, using a simple easy to use scheme called the Leicester Grading System (Fig. 2). It has been shown to predict BCVA in adults^16^ as well as vision later in life in young children.^15^ Grade 1 of the scheme corresponds to mild FH and grade 4 to the most severe FH, where the foveal area is similar to peripheral retina (fovea plana). Mild forms of FH can be further distinguished into grade 1a and 1b depending on the appearance of the foveal pit.^20^ Kuht et al. have recently shown that oculocutaneous albinism is associated with all grades of FH,^4^ whereas ocular albinism and Foveal Hypoplasia, Optic Nerve Decussation defect, and Anterior segment abnormalities syndrome (FHONDA)^29^ are associated with severe FH (grades 3 and 4) despite not manifesting obvious pigmentation deficits.

In the Leicester Grading System, each grade is associated with a relatively wide range of BCVAs with Kuht et al.^4^ reporting interquartile ranges of 0.3 logMAR or more for grades 2 to 4, the most common grades associated with albinism, in 597 participants with genes associated with typical hypoplasia, of which 67.5% had albinism (Fig. 1D). Although the scheme is clinically useful, it limits a more precise predictive potential of the system for BCVA as other visual anomalies could contribute to variability in BCVA for each grade of FH which are not taken into account. One such anomaly is nystagmus.

## Effect of Nystagmus on BCVA in PwA

Nystagmus exists in most people with PwA.^18^^,^^30^ It is typically horizontal, conjugate, with a combination of jerk and pendular waveforms and is similar in appearance to people with idiopathic infantile nystagmus (IN) who normally have less severe FH.^9^ Nystagmus can usually be modulated in PwA by moving the gaze away from or toward the null region where the nystagmus is less intense. As part of: (i) a randomized controlled trial (RCT) comparing optical treatments for infantile nystagmus,^31^ and (ii) a large, as yet unpublished, pharmacological RCT (McLean RJ, et al. IOVS 2016;57(12): ARVO Abstract) we characterized the foveal hypoplasia in 22 participants with albinism (mean age ± SD = 30.6 ± 11.5 years; FH grades 1, 2, 3, and 4: *n* = 2, 4, 8, and 8, respectively) along with 20 participants with idiopathic IN mean age ± SD = 33.8 ± 12.7 years; (no FH: *n* = 14; FH grades 1 and 2: *n* = 5 and 2, respectively).

**Figure 3.**
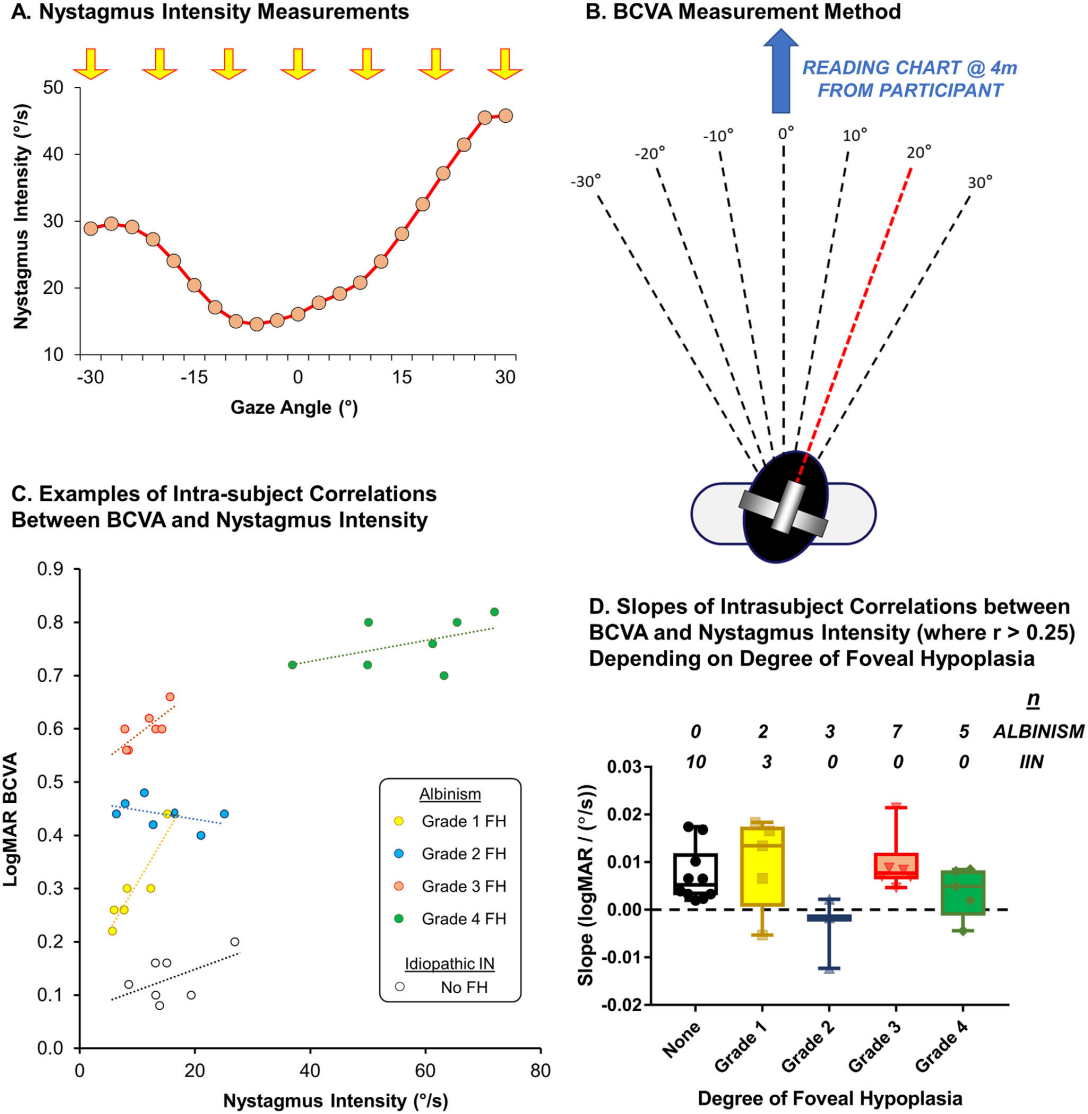
Previously unpublished findings showing the comparison of within subject changes in best-corrected visual acuity (BCVA) with nystagmus intensity in albinism and idiopathic Infantile Nystagmus. In (**A**) calibrated nystagmus traces, recorded using an EyeLink II eye tracker (SR Research, Ottawa, Canada), were used to derive nystagmus intensity (amplitude × frequency) across the horizontal axis from −30 degrees (to the left) to +30 degrees (to the right). Nystagmus intensity values were taken at −30 degrees, −20 degrees, −10 degrees, 0 degrees, +10 degrees, +20 degrees, and +30 degrees gaze angles and compared to (**B**) BCVAs recorded at the same gaze angles using an electronic PVAAT visual acuity tester (EDTRS charts; Precision Vision, Woodstock, Illinois, USA). Examples of intrasubject scatter plots between nystagmus intensity and BCVA with best-fit lines are shown in (**C**). Slopes for the best-fit lines for each participant in which the *r* value were > 0.25 are shown in (**D**). Within grade 1 are 3 participants with grade 1a and 2 participants with grade 1b.

**Figure 4.**
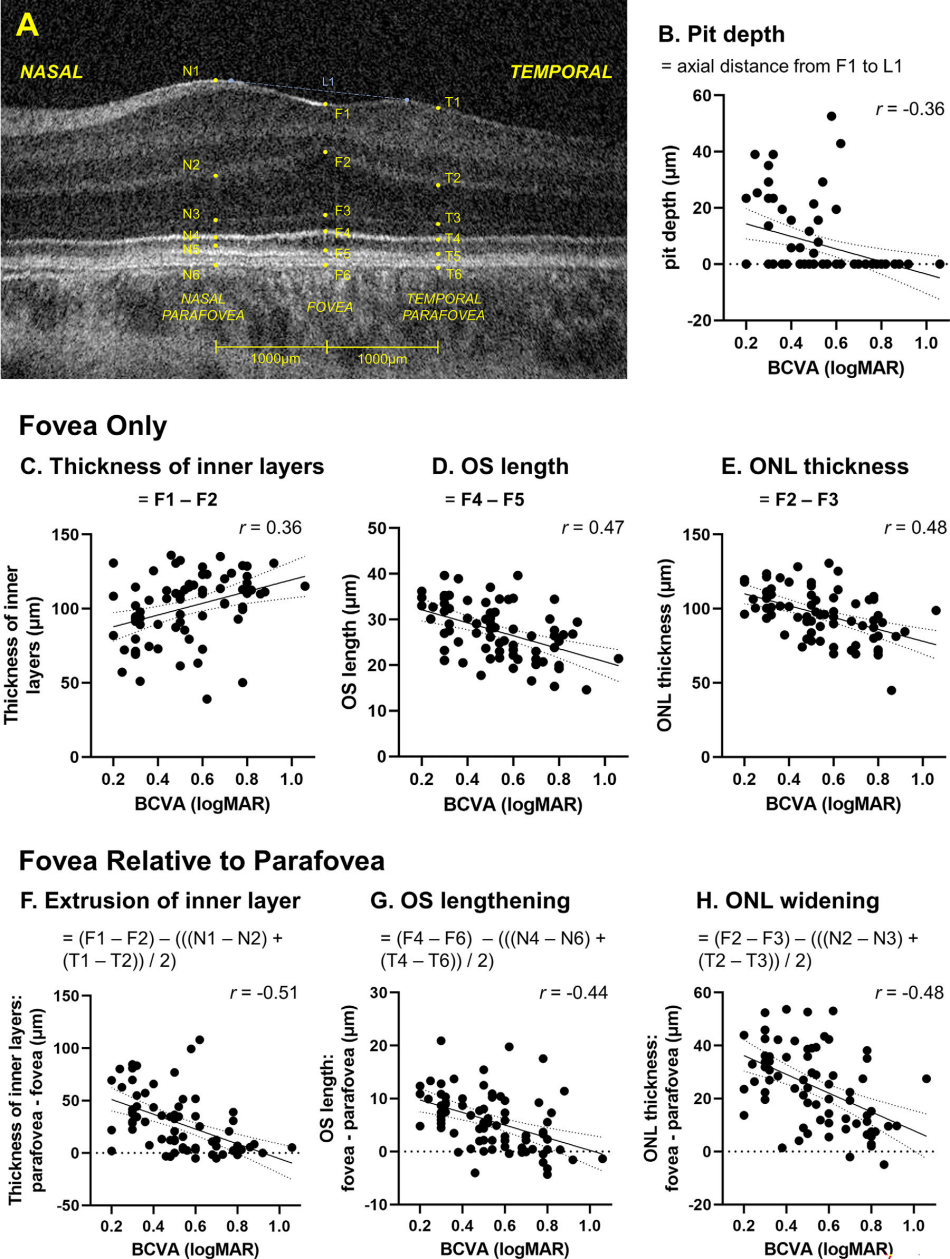
Previously unpublished findings showing associations between objective measures of signs used to grade foveal hypoplasia with the Leicester Grading Scheme and best-corrected visual acuity (BCVA). (**A**) provides a key indicating how the measures were derived from 74 adult participants with albinism on an optical coherence tomography b-scans through the center of the fovea. (**B**) Shows the depth of the foveal pit where no pit is indicated by a zero value. Measures through the center of the fovea are shown for: (**C**) thickness of inner layers, (**D**) photoreceptor outer segment (OS) length, and (**E**) outer nuclear layer (ONL) thickness. The same 3 measures are then presented in (**F**) to (**H**) with respect to the difference between the fovea and parafoveal visions measured at 1000 µm either side of the fovea (average of nasal and temporal). Foveal measurements: F1 = internal limiting membrane, F2 = border of outer plexiform layer and ONL, F3 = external limiting membrane, F4 = center of the ellipsoid, F5 = cone outer segment tips (COST), and F6 = Bruch's membrane. N1 to N6 and T1 to T6 are the equivalent measures for the nasal and temporal parafovea, respectively. Bruch's membrane (N6, F6, and T6) was used as a reference for (**G**) because this border is easy to distinguish compared to COST in the parafovea. OCT images were used from eyes with the best quality images for analysis, or from right eyes if image quality was similar (*n* = 44 right eyes and *n* = 33 left eyes). BCVAs were recorded with both eyes open.

At baseline visits, the same approach was used in both studies, where nystagmus and BCVA were recorded while participants attempted to hold gaze at 10-degree intervals from 30 degrees to the left to 30 degrees to the right. Nystagmus intensity was calculated from calibrated eye movement recordings (Fig. 3A) and BCVA measured using an electronic reading chart which allowed randomization of Early Treatment Diabetic Retinopathy Study (ETDRS) optotypes (Fig. 3B). Large single frame glasses were provided to each participant, which we previously demonstrated were not detrimental to BCVA measures in lateral gaze compared to hard contact lens wearing where the lenses move with the eyes.^31^ Best-fit lines of within subject correlations between nystagmus intensity and BCVA were used to estimate the effect of nystagmus on BCVA (Fig. 3C). The methodologies used to record, calibrate, and analyze eye movements and measure BCVAs are described in full in our study Jayaramachandran et al. (2014)^31^ (for example, see Fig. 4 which illustrates the approach used on one participant). The same methodology has been used throughout in the unpublished RCT by McLean RJ, et al. (IOVS 2016;57(12): ARVO Abstract).

In participants demonstrating a significant correlation between nystagmus intensity and BCVA (r > 0.25: *n* = 17 PwA and *n* = 13 idiopathic IN) a wide range of slopes were observed (Fig. 3D) mostly in the positive direction. There was no significant relationship between slopes for each individual and FH grade (*P* = 0.41) indicating that degree of FH does not significantly affect the role that nystagmus has in modifying BCVA. Including all data into linear mixed models showed that nystagmus intensity was strongly associated with BCVA in both PwA and idiopathic IN (*P* < 0.0001). However, parameter estimates for nystagmus intensity were low at 0.0032 for PwA (95% confidence interval [CI] = 0.0016–0.0048) and 0.0046 for idiopathic IN (95% CI = 0.0030–0.0062) predicting that abolishing a typical nystagmus of 20 degrees/second intensity would only improve vision by only 0.064 and 0.092 logMAR, respectively.

These data indicate that nystagmus is not the only factor influencing BCVA even after FH is taken given the wide interquartile ranges in BCVA for moderate and severe foveal hypoplasia. Similar conclusions can be drawn for mild or no FH, which consolidates the findings of other groups investigating the effect of nystagmus in idiopathic IN on BCVA.^32^^–^^34^

These data also suggest that therapeutic studies aiming to reduce nystagmus are likely to have limited efficacy in improving BCVA. However, several individuals in Figure 3D demonstrated slopes of 0.01 or higher, although mostly in lower grades of FH, which was more commonly associated with idiopathic IN rather than albinism. Abolishing a typical nystagmus of 20 degrees/second in these individuals has the potential of improving vision by 0.2 logMAR lines and identifying these individuals could offer a personalized approach to potential treatments for nystagmus. BCVAs were measured in this experiment after short-term changes in nystagmus intensity and possibly therapies causing long-term changes in nystagmus intensity could cause bigger changes in BCVA, for example, through neural plasticity.

## Associational Relationships Between Anomalies in the Visual Pathway and BCVA in PwA

In a series of studies, we have characterized anomalies throughout the visual system in PwA, which has also enabled us to investigate the association between abnormalities at various stages of the visual pathway and their relationship to BCVA (Table 1).^2^^,^^5^^,^^6^^,^^9^^,^^17^^,^^35^ Of the visual anomalies investigated, foveal hypoplasia, nystagmus, and iris pigmentation were the phenotypical features that generated the strongest associations to BCVA. Including outer segment length, nystagmus intensity and posterior epithelial layer (PEL) thickness into a single linear regression model in 36 participants improved the *r* value to 0.783 (95% CI = 0.612 to 0.884) explaining 61.3% of the variance in BCVA compared to individual factors which explained no more than 30% of the variance. Using FH graded using the Leicester Grading System instead of outer segment length also yielded an *r* value of 0.777 (95% CI = 0.602 to 0.881) explaining 60.4% of the variance (see Table 1). Significant associations do not necessarily indicate causality, especially in albinism where these may simply be due to the overall degree of albinism. However, these measures relate to three key phenotypical features that one would expect to impact upon visual acuity at a retinal level, (i) the degree of foveal hypoplasia, (ii) the amount of retinal motion caused by nystagmus, and (iii) hyper-illumination of the eye caused by iris transillumination.

**Table 1.** Results of Linear Modelling to Explore How Much Variance in Best-Corrected Visual Acuity (BCVA) Is Explained by Measures of Visual Anomalies From Various Studies in Adult Participants With Albinism.

Methodology/Source of Data	Parameter	*n*	*r* [95% CI]	*P* Value	Variance Explained
**Foveal measurements (posterior segment OCT)**					
Posterior segment OCT data acquisition described in Mohammad et al., 2011^17^ OCT data collected for PhD theses of Sarim Ather and Viral Sheth. See Figures 2 and 4 for analysis	Foveal hypoplasia grading	**74**	**0.528 [0.341 to 0.675]**	**0.000001**	**27.88%**
	Thickness of photoreceptor layers	**74**	**−0.530 [−0.676 to −0.343]**	**0.000001**	**28.05%**
	Outer nuclear layer thickness	**74**	**−0.512 [−0.663 to −0.321]**	**0.000003**	**26.22%**
	PR inner segments length	74	**−**0.068 [**−**0.292 to 0.163]	0.578	0.46%
	PR outer segments length	**74**	**−0.522 [−0.671 to −0.333]**	**0.000003**	**27.25%**
**Optic nerve head changes (posterior segment OCT)**					
^*^Posterior segment OCT data acquisition and analysis + correlation with BCVA described in Mohammad et al., 2015^5^	Disc area	**52**	**−0.386 [−0.596 to −0.126]**	**0.005**	**14.90%**
	Rim area	52	**−**0.253 [**−**0.492 to 0.0214]	0.071	6.40%
	Cup-to-disc ratio	52	**−**0.121 [**−**0.381 to 0.157]	0.395	1.46%
	cpRNFL thickness	46	0.052 [**−**0.224 to 0.320]	0.731	0.27%
**Retrobulbar measures (MRI and VEP)**					
MRI (structural and DTI) acquisition and analysis described in Ather et al., 2019^6^ VEP acquisition and analysis described in PhD Thesis, Sarim Ather; Correlation of optic nerve, tract and chiasmal cross-sectional areas to BCVA found in PhD Thesis of Sarim Ather^†^	Optic nerve cross sectional area	22	0.205 [**−**0.237 to 0.577]	0.350	0.92%
	Optic tract cross sectional area	22	0.010 [**−**0.414 to 0.429]	0.919	0.01%
	Chiasm cross sectional area	22	0.010 [**−**0.414 to 0.429]	0.848	0.20%
	Tractography of chiasm – SDI	22	0.010 [**−**0.414 to 0.429]	0.051	18.66%
	Occipital pole thickness	22	0.108 [**−**0.329 to 0.506]	0.633	1.16%
	Occipital pole volume	22	0.257 [**−**0.185 to 0.612]	0.249	6.59%
	VEP asymmetry	44	0.035 [**−**0.265 to 0.328]	0.823	0.12%
**Nystagmus (eye movement recordings)**					
Eye movement data acquisition and analysis described in Jayaramachandran et al., 2014^32^ Kumar et al., 2011^9^ and McLean et al., 2007^36^	Amplitude	**61**	**0.444 [0.216 to 0.626]**	**0.0003**	**19.71%**
	Frequency	61	0.085 [**−**0.170 to 0.330]	0.509	0.72%
	Intensity	**61**	**0.451 [0.225 to 0.631]**	**0.0002**	**20.34%**
	NAFX	**61**	**0.424 [0.193 to 0.611]**	**0.0004**	**17.98%**
**Iris structure and pigmentation (anterior segment OCT and iris appearance)**					
^*^Anterior segment OCT acquisition and analysis + correlation with BCVA described in Sheth et al., 2013^2^	TID grading	55	0.206 [**−**0.0627 to 0.447]	0.132	4.24%
	PEL thickness (all)	**55**	**−0.416 [−0.614 to −0.169]**	**0.002**	**17.31%**
	PEL thickness at root	**55**	**0.400 [0.151 to 0.602]**	**0.001**	**20.52%**
**Visual fields (Humphrey visual field testing)**					
^‡^Humphrey VF testing and analysis described in Sheth et al., 2024^42^	Whole visual field	**62**	**0.491 [0.275 to 0.660]**	**0.000001**	**24.13%**
	Central visual field	**62**	**0.471 [0.250 to 0.645]**	**0.0000002**	**22.14%**
	Peripheral visual field	**62**	**0.453 [0.229 to 0.631]**	**0.00001**	**20.52%**
**Eye biometry (ultrasound and refractive assessment)**					
A-scan ultrasound measurements and refractive assessment described in PhD thesis, Viral Sheth^†^	Axial length	42	0.262 [**−**0.0456 to 0.524]	0.094	6.86%
	Spherical equivalent	41	0.306 [**−**0.00188 to 0.561]	0.052	9.36%

cpRNFL, circumpapillary retinal nerve fiber layer; OCT, optical coherence tomography; PEL, posterior epithelial layer; PR, photoreceptors.

Photoreceptor layers include all retinal layers at the fovea from retinal pigment epithelium to the outer nuclear layer/outer plexiform layer border.

Nystagmus intensity was calculated from the mean peak to peak amplitude times the frequency of the nystagmus. NAFX (eXpanded Nystagmus Acuity Function), a measure of nystagmus foveation, was estimated using the MatLab code developed by the Dell'Osso laboratory (www.omlab.org).

The formula developed by Apkarian was used to determine visual evoked potentials (VEP) asymmetry.^57^

Iris transillumination defects (TID) were graded using the system devised by Summers.^3^

Significant predictors of BCVA are highlighted in bold.

All data are BCVAs with both eyes viewing and mean measures from left and right eyes. Exceptions are foveal measurements (see legend of Fig. 4) and visual fields where p values are generated using linear mixed models.

* Indicates data including correlations with BCVA already published.

† PhD these available at University of Leicester Institutional Repository, UK.

‡ This study is currently under review at IOVS. Revisions have been submitted. Please see the attached manuscript for the latest draft.

Later stages of the visual pathway beyond the retina are also likely to determine BCVA in albinism. In the normal visual system, foveal cone packing densities reflect BCVA because of 1:1 cone-to-retinal ganglion cell ratios preserving parvocellular parallel line of sensory inputs into the cortex and cortical magnification of the foveal region in area V1 and beyond.^36^ Recently, Woertz has shown that cortical magnification in PwA is more accurately predicted by retinal ganglion cell density rather than cone density suggesting that decreased foveal cone density in PwA may be partially counteracted by changes in downstream connectivity.^37^ Interestingly, visual field measures were strongly correlated with BCVA (see Table 1) which, similar to BCVA, are psychophysical measures of vision that capture deficits along the whole visual pathway.

Our data indicates that hypopigmentation of the iris and fundus, is also likely to influence BCVA. The detrimental effect on vision could be caused by glare due to increased light passing through the iris to the retina. However, glare is not easy to evaluate clinically in PwA.

## Improving Prediction of BCVA in PwA

In summary, the current FH grading provided by the Leicester Grading System provides a rapid and easy to use approach to assess the degree of FH in the clinic, and hence has widespread applicability. However, each grade of FH is associated with a relatively wide range of BCVAs, especially the FH grades found in albinism. Although this is helpful clinically, including nystagmus and iris pigmentation measures, along with the genetic origin of albinism, could potentially improve future prediction models of BCVA in PwA.

A limitation with the Leicester Grading System is that it takes a binary approach to the appearance of foveal hypoplasia characteristics in order to allow rapid classification, where: (i) extrusion of inner retinal layers, (ii) foveal pit, (iii) outer nuclear layer (ONL) thickening, and (iv) OS lengthening, are either judged to be absent or present (grades 1a and 1b are distinguished by either a well-formed or shallow / absent foveal pit). In reality, these signs form a continuous and partially overlapping spectrum, as shown in Figure 4, which includes data from 74 PwA (see Table 1 for details of data collection), where, pit depth (Fig. 4B), thickness of inner retinal layers (Fig. 4C), OS length (Fig. 4D), and ONL thickness (Fig. 4E), are all negatively or positively correlated with BCVA changing concurrently with the degree of visual deficit.

In Figures 4F–H, the difference in measurements at the fovea expressed in relation to the parafovea are shown for inner retinal layers (see Fig. 4F), OS length (see Fig. 4G), and ONL thickness (see Fig. 4H), where a value of zero indicates that there is no specialization at the fovea with respect to the parafovea. As predicted by the Leicester Grading System, the general trend in PwA with increasingly poorer vision is for the pit to disappear first (see Fig. 4B), followed by the loss of OS lengthening (see Fig. 4G), and then absence of ONL widening (see Fig. 4H). All PwA included in the sample show continuity of inner retinal layers across the fovea (see Fig. 1C). but the difference between the thickness of inner retinal layers in the fovea compared to the parafovea becomes increasingly smaller as vision gets worse (see Fig. 4F). Recent studies have shown that in albinism carriers, changes in the inner retina occur in the absence of changes in the outer retinal layers compared to normals,^28^^,^^38^ confirming the use of extrusion of inner plexiform layers as an effective way to distinguish low-grade FH (grade 1a) to no FH at all.

Combining either foveal parameters (pit depth, thickness of inner retinal layers, OS length, and ONL thickness) or parameters indicating specialization of the fovea in respect to the parafovea (pit depth, extrusion of inner layers, OS lengthening, and ONL widening) into linear regression models only improved prediction of BCVA slightly compared to individual measures (*r* = 0.535, 95% CI = 0.350 to 0.681 and *r* = 0.553, 95% CI = 0.372 to 0.694, respectively, compared to *r* values on Figs. 4B–H).

A further development of quantifying the structural features of foveal hypoplasia would be to generate objective criteria for FH grading, as is currently being developed by Woertz et al. 2020 (Woertz EN, et al. IOVS 2020;61:5261, ARVO Abstract). This could incorporate additional features that are currently not captured with subjective grading. One such feature, is the asymmetric distribution of the ganglion cell layer (GCL) around the fovea in albinism due to thicker GCL on the nasal compared to the temporal aspect.^39^^–^^41^

## Interocular Asymmetries in BCVA in PwA

Another aspect of BCVA that has not been fully investigated in PwA is the difference in BCVA between the two eyes in relation to amblyogenic factors in PwA. In a recent study documenting visual field deficits in PwA, we observed very similar BCVAs between the two eyes despite a clear interocular asymmetry in visual fields which were significantly worse in left eyes compared to right eyes.^42^ The similarity in the BCVA of the two eyes was surprising given that albinism is known to be associated with amblyogenic factors.^9^ To investigate this issue further, we accurately assessed amblyogenic factors in PwA without amblyopia.

**Figure 5.**
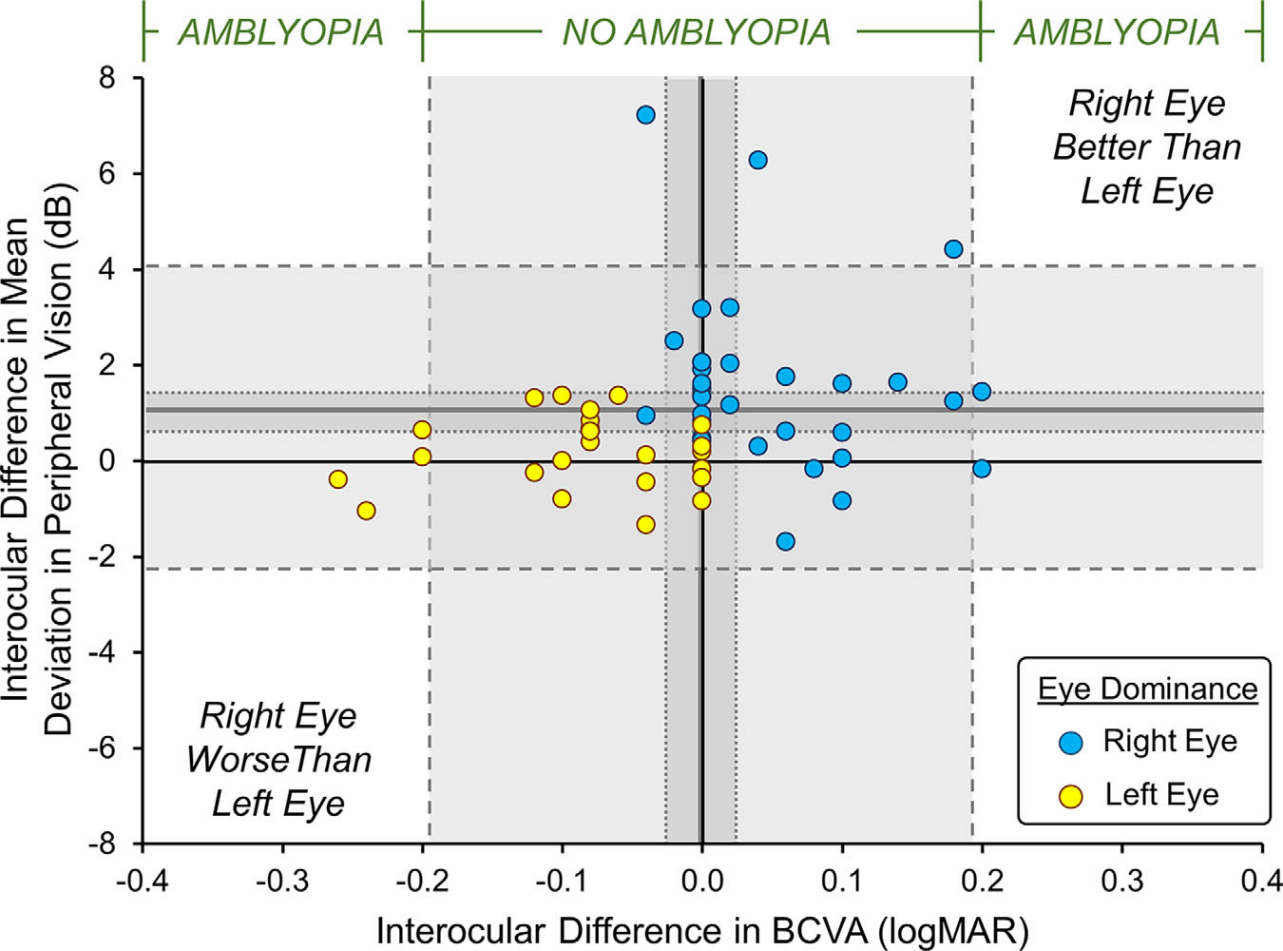
Previously unpublished findings showing correlation of interocular asymmetries in central (BCVA) and peripheral vision (peripheral visual field quadrants) in 61 people with albinism (PwA) (see the attached document [the manuscript is currently under consideration at IOVS] for further details on data collection). Interocular asymmetries are plotted for right eyes (*blue circles*) and left eye dominant (*yellow circles*) individuals where positive values indicate better vision in the right eyes.

Figure 5 shows the correlation between central vision (i.e. in BCVA) and peripheral vision (average of 4 peripheral visual fields quadrants) interocular asymmetries in 61 PwA with different symbols representing left (yellow circles) and right eye (blue circles) dominant individuals. Overall, there was a low incidence of amblyopia as defined by an interocular difference of > 0.2 logMAR BCVA between the 2 eyes (see Fig. 5). The 95% prediction intervals for the interocular asymmetry in BCVA all fell below the definition of amblyopia with only 2 participants (3.3%) that demonstrated amblyopia (Table 2). Only 18.0% of participants demonstrated an interocular difference of > 0.1 logMAR. Strabismus and anisometropia was measured in 39 PwA who had equal or near equal vision (< 0.1 logMAR interocular difference; see Table 2). Of these, 84.6% demonstrated one or more amblyogenic factor.

**Table 2.** Previously Unpublished Findings Showing Proportion of Amblyopia and Amblyogenic Findings in People With Albinism (PwA)

Interocular Difference in BCVA	*n*	%
**Proportion of amblyopia**		
>0.2 logMAR	2	3.3%
>0.1 logMAR	11	18.0%
**No amblyopia**		
≤0.1 logMAR	50	82.0%
equal vision	21	34.4%
total	61	
**Amblyogenic factors in PwA with near or equal vision (<0.1 logMAR)**		
Strabismus	28	71.8%
Anisometropia	7	18.0%
Either	33	84.6%
Total	39	

BCVA, best-corrected visual acuity; LogMAR, logarithmic Minimum Angle Resolution.

Note: See the attached document of the manuscript currently under consideration at IOVS. (A) The proportion of PwA (*n* = 61) with interocular differences in visual acuity, and (B) proportion of PwA who demonstrated amblyogenic factors who did not have amblyopia (*n* = 39).

This was mainly due to strabismus, which was measurable in 71.8% of participants (nystagmus can mask a small strabismus, hence 71.8% is likely to be an underestimate), with anisometropia (≥ 1 diopter difference between the eyes) occurring in 18.0% of participants.

In the normal visual system, equivalent areas in the visual fields arising from left and right eye inputs are brought together in adjacent ocular dominance columns of the primary visual cortex providing the neural basis of retinal disparity cues for stereopsis. This sets up the possibility of an interaction between visual field representations for the left and right eyes. In conventional amblyopia, decorrelated inputs from left and right eyes, caused by strabismus and/or anisometropia, lead to altered excitatory/inhibitory balance of the two eyes at the local circuit level.^43^^,^^44^ In PwA, chiasmal misrouting disrupts the normal pattern of ocular dominance columns, which are replaced by partial representations of the ipsilateral visual hemifield being superimposed onto the normal contralateral hemifield representations.^10^^–^^14^ This prevents the possibility of parts of the visual input for the left and right eyes interacting with each other which may not cause the cortex to suppress inputs from one eye.

Here, we have focused on the potential effect of albinism on interocular differences in visual acuity, that is, monocular amblyopia, however, it is possible that some individuals with albinism might present with bilateral amblyopia. Visual deprivation early in life, for example, due to infantile cataracts, can lead to profound unilateral or bilateral vision loss. Recent studies investigating retinal development in PwA in early years using handheld OCT have shown, not just FH, but a delay in the time course of foveal development in PwA compared to normal, especially of the outer retina.^45^ It is possible that such a delay in foveal development could cause bilateral amblyopia. In addition, nystagmus is also known to be associated with meridional amblyopia.^46^^,^^47^

## Concluding Remarks

Retinal imaging has greatly advanced our understanding of the relationship between foveal structure and BCVA in PwA but including additional factors, such as nystagmus and hypopigmentation / glare, into statistical models in addition to foveal hypoplasia could improve prediction. Interestingly, visual acuity deficits are similar in the two eyes of PwA, presumably because ocular dominance columns in the primary visual cortex have been replaced with hemifield dominance columns.

One issue that needs to be borne in mind is that BCVA is a limited aspect of vision that does not reflect the full visual deficit associated with albinism. Other important features of vision include:

• **Vision across the range of gaze angles used by an individual:** Although head postures are frequently adopted by PwA to optimize the use of the null region, PwA will often move their eyes away from the null region during visual search. Attempts have been made to capture the deficit across a region of gaze angles using a gaze-dependent visual acuity task similar to the method described in Figure 1B.^31^^,^^34^^,^^48^
• **Time-to-see:** Investigations have indicated that people with nystagmus take longer to get their eyes on a target, a situation that is likely to be made worse in PwA who are likely to have more severe FH compared to other types of nystagmus.^4^ Attempts have also been made to measure time-to-see clinically using a time-restricted visual acuity measurement.^48^
• **Stereovision** is affected in PwA because of chiasmal misrouting who show little or no stereopsis.^9^
• **Contrast Sensitivity** is known to be reduced in PwA.^49^^,^^50^
• **Motion Sensitivity:** Elevated motion thresholds are reported in PwA.^51^
• **Peripheral vision:** Few studies have investigated deficits in peripheral vision in PwA. Psychophysical studies have indicated that people with infantile nystagmus do not appear to have difficulties in locating targets in space presented in peripheral vision indicating that the efference copy of the oculomotor system is likely to be effective despite involuntary eye movements caused by nystagmus.^52^^,^^53^ PwA have also been reported as having contracted visual fields measured using kinetic perimetry. We have recently used static perimetry to demonstrate reduced detection thresholds across the visual field but especially in the superior nasal quadrant (this study is also found in this special IOVS issue on albinism).^42^
• **Integration of foveal and parafoveal vision:** Reading is a skill that requires integration of foveal and parafoveal information. Previous studies indicate that PwA can read reasonably quickly provided that optimal font sizes are provided.^54^ However, reading speeds are often suboptimal, for font sizes, a number of lines worse than that predicted by BCVA measures, possibility because of poorer integration of foveal and parafoveal vision.^54^

One of the pertinent issues in relation to some of these functional aspects of vision is that they are not fully captured adequately in the clinic, or that the relationship between functional vision and standard clinical measures, such as BCVA, stereoacuity, and orthoptic assessments, OCT imaging of the retina and eye movement recordings, are poorly understood. New measurement tools are under development for use in the clinic to address this issue.^55^
